# Determinants of dietary behaviors among dental professionals: insights across educational levels

**DOI:** 10.1186/s12903-024-04502-4

**Published:** 2024-06-24

**Authors:** Athikom Tantimahanon, Kawin Sipiyaruk, Chanita Tantipoj

**Affiliations:** 1 Department of Advanced General Dentistry, Faculty of Dentistry, Mahidol University, 6 Yothi Road, Ratchathewi, Bangkok, 10400 Thailand; 2 Department of Orthodontics, Faculty of Dentistry, Mahidol University, 6 Yothi Road, Ratchathewi, Bangkok, 10400 Thailand

**Keywords:** Dental education, Dental professionals, Dietary behaviors, KAP, Eating behavior, Well-being

## Abstract

**Background:**

Healthy dietary behaviors are fundamental for maintaining optimal health. Understanding the dietary behaviors of dentists is vital for designing effective interventions to foster healthier behaviors. However, investigations into dietary behaviors and their associations among dental professionals have been limited.

**Objective:**

To investigate the dietary behaviors of dental professionals, along with the associated factors influencing their dietary options.

**Materials and methods:**

A self-administered online questionnaire was constructed to collect data from three groups of dental professionals: undergraduates (UG), postgraduates (PG), and practicing dentists (DT). The questionnaire encompassed inquiries regarding demographic characteristics, knowledge assessment, evaluation of attitudes, and examination of dietary behaviors. Data analysis procedures included descriptive statistics, Spearman’s rank correlation, and multiple linear regression.

**Results:**

A total of 842 individuals participated in the study (UG: 264, PG: 247, DT: 331). Attitude emerged as the strongest association of healthy dietary behaviors across all groups (UG: ß=0.370, PG: ß=0.512, DT: ß=0.642; *P* < 0.001), while alcohol consumption showed a negative correlation with healthy dietary behaviors (UG: ß=-0.135, PG: ß=-0.220, DT: ß=-0.216; *P* < 0.001).

**Conclusion:**

Significant variations in dietary behaviors across diverse educational levels of dental professionals were observed. Attitude emerged as the predominant factor influencing dietary behaviors, while knowledge was found to have a weak association. Tailored interventions addressing individual challenges at different career stages should be considered to enhance dietary behaviors and overall well-being in dental practice settings.

**Supplementary Information:**

The online version contains supplementary material available at 10.1186/s12903-024-04502-4.

## Introduction

Dietary behaviors play an important role in human life, exerting significant influence on health outcomes and susceptibility to various illnesses [1]. These behaviors could lead to non-communicable diseases, such as cardiovascular disease, diabetes and cancer, which are major contributors to global mortality [2]. Furthermore, available evidence demonstrates the association between dietary behaviors and oral health [3]. Studies have revealed that eating disorders can have a significant impact on oral health [4–6]. Additionally, dietary and physical activities have shown higher effectiveness and benefits compared to pharmacological interventions, considering risks, side effects, and expenses [7, 8]. Various epidemiological studies emphasize and provide recommendations for the prevention and treatment of chronic diseases.

A number of internal and external factors can impact dietary behaviors. These factors encompass various aspects, including nutrition knowledge, the availability of different food options, individual attitudes toward diet, emotional well-being, personal experiences, and the socio-cultural environment in which eating occurs [9]. Medical professionals, such as primary care doctors and nurses, play a critical role in assisting patients in adopting healthier behaviors to reduce the risk of developing diseases. Promoting better dietary behaviors is a means to enhance physical health [10, 11]. Dental professionals also play a vital role in the healthcare system by assisting patients in modifying their diet to improve overall health. Nutritional guidance from dentists not only benefits patients’ physical well-being but also positively impacts their oral health [12].

Dental professionals appear to encounter various health issues, such as musculoskeletal and stress-related problems. This is primarily due to the demanding nature of their work, which necessitates coordination between their eyes and other bodily components [13]. Dentists’ health and well-being can be impacted by various factors, including personal, professional, job-related, workplace-related, healthcare system-related, and regulatory factors [14]. Additionally, a survey on health behaviors among dentists revealed concerning trends, such as high alcohol consumption and medication usage for cholesterol and blood pressure management [15]. Despite awareness of the adverse effects of sugary foods on dental health [16], previous studies have found that dentists are more likely to consume sugary foods between meals [17–19]. Understanding the dietary behaviors and determinants of dentists is essential for crafting effective strategies to foster healthier eating habits among them, which can consequently benefit their patients. Studies have shown that physicians possess the ability to influence patients’ health behaviors through preventive advice and the promotion of healthy habits. The likelihood of physicians providing counseling on health promotion is closely tied to their own health practices [11]. Therefore, addressing the health behaviors of healthcare providers themselves is crucial for significantly enhancing health promotion counseling in primary care settings.

The Knowledge, Attitude, and Practices (KAP) model has commonly been used to elucidate individual health behavior [20]. Recent evidence has established connections between knowledge, attitude, and practices [21]. While most research suggests that higher knowledge correlates with greater adoption of preventive measures and positive attitudes are associated with preventive behaviors [22], several studies have indicated a weak or no association between knowledge and practices [23–25]. Additionally, social and environmental determinants have been identified as key factors influencing individual health behaviors [26, 27]. A study evaluating dietary behaviors among dental students recommended integrating attitudes and enthusiasm for nutrition concepts into the curriculum to enhance knowledge and practices, potentially leading to improved oral health outcomes for patients [28]. Given that attitudes appear to be a crucial determinant influencing practices, variations in dietary behaviors among different educational levels of dental professionals could exist.

The degree of involvement in the dental profession may have potential to impact dietary behaviors. Specifically, among dental undergraduates, postgraduates, and professionals with varying contexts to their practices, diverse dietary behaviors may emerge. Previous studies investigating dental professional behaviors often categorized participants into only one or two groups of dental undergraduates, postgraduates, or professionals [17, 28, 29]. However, research incorporating data from all three groups was limited. Examining the dietary behaviors of dentists according to their level of education can be supportive in developing strategies to promote positive behaviors, thereby enabling dental professionals to effectively encourage patients to adopt healthier behaviors. Therefore, the aim of this study was to investigate the dietary behaviors of dental professionals, along with the associated factors influencing their dietary options. It was hypothesized that dietary behaviors among dental professionals vary according to their level of education.

## Materials and methods

### Research design

This study employed a cross-sectional research design using an online questionnaire survey to gather data from dental students and professionals in Thailand. An online questionnaire was administered between April and October 2023. The ethical approval for the study was certified by the Institutional Review Board of Faculty of Dentistry and Faculty of Pharmacy, Mahidol University, the ethical approval number: MU-DT/PY-IRB 2023/004.1701.

### Research participants

The study population comprised three distinct groups: 4th to 6th year dental undergraduates (UG), postgraduates (PG), and dentists (DT). The inclusion criteria for dental undergraduates and postgraduates were individuals with valid registration at dental schools in Thailand. Additionally, dentists holding a license from the Thai Dental Council were included. Exclusion criteria included dental students who had discontinued their studies and dentists not actively practicing in the field.

The sample size for this study was estimated using the formula for a finite population [30]. With the presumption of a 95% confidence interval coupled with a 6% margin of error, this study expected to recruit 245 undergraduates, 235 postgraduate students, and 269 dentists, resulting in a total sample size of 749 participants across all three groups of dental professionals.

### Questionnaire construction

The questionnaire was developed based on previous literature focusing on determinants related to dietary behaviors [31, 32]. The questionnaire consisted of four parts: Part 1: Demographic data (personal, work-related, and environmental information), Part 2: Knowledge related to dietary behaviors. Scoring criteria was that one point was assigned for a correct response, and no points were allocated for an incorrect or unknown answer, Part 3: Attitude toward dietary behaviors. This section utilized a 5-point Likert scale, ranging from ‘strongly disagree’ to ‘strongly agree’ and Part 4: Dietary behaviors. This section also employed a 4-point Likert scale, ranging from ‘never’ to ‘always’. The questionnaire was administered online via Google Forms. The link to the questionnaire was initially distributed to twelve representatives, who were instructed to further disseminate it through group chats of a messenger application among dental student, resident, and alumni networks.

Validity and reliability were examined to ensure the quality of the questionnaire. Content validity was assessed by three experts, including two dental education specialists (one with a doctoral degree in education and the other with significant publications in educational research) and one advanced general dentistry specialist. The Item-Objective Congruence (IOC) statistic was utilized for evaluating the content of each question. The questions were iteratively revised until each item achieved an IOC value higher than 0.5. The validated questionnaire was piloted with a sample of 30 respondents, comprising 10 dental undergraduates, 10 postgraduates, and 10 dentists. The questionnaire was distributed to a pilot group with a two-week interval. Test-retest reliability was initially evaluated using the Kappa statistic, with a minimum value set at 0.70. Additionally, Cronbach’s Alpha coefficient was employed to measure internal consistency reliability. The coefficients for the knowledge, attitude, and practice constructs were 0.70, 0.73, and 0.75, respectively.

### Data analysis

The data management and analysis process involved using STATA software, version 18. Descriptive statistics were employed to determine response frequencies and elucidate sample characteristics. The Pearson’s chi-square test was used to assess correlations among demographic data across groups of dental professionals. Spearman’s Rank Correlation was applied to investigate potential associations between continuous variables, such as knowledge, attitude, and behavior scores. Multiple linear regression was employed to identify determinants of healthy dietary behavior outcomes. All variables, including demographic data, knowledge scores, and attitude scores, were included in the analysis, with practice score serving as the dependent variable. Statistical significance was set at a *p*-value less than 0.05.

## Results

### Research participants

This survey included a total of 842 individuals, with 264 in the UG group, 247 in the PG group, and 331 in the DT group. Across all groups, a majority of participants were female (UG: 67.1%, PG: 77.7%, DT: 72.2%), and most were younger than 30 years old (UG: 98.1%, PG: 87.5%, DT: 59.2%). Additionally, a significant portion of participants in all three groups report no history of medical diseases (UG: 83.0%, PG: 75.3%, DT: 75.8%) and maintain a normal weight (UG: 64.4%, PG: 69.2%, DT: 68.0%). Table 1 presents the general characteristics of the participants, categorized by their level of dental education.

More than half of the respondents indicate drinking alcohol less than once per month (UG: 64.8%, PG: 57.5%, DT: 53.2%), and approximately half report engaging in 150 min or less of weekly exercise (UG: 49.6%, PG: 53.4%, DT: 53.5%). The majority of participants in the UG and PG groups report sleeping less than 7 h per night, with rates of 60.6% and 72.9%, respectively. In contrast, only 40.5% of the DT group report sleeping less than 7 h per night. Interestingly, less than half of UG participants (44%) report practicing dentistry for about 21–30 h per week, whereas 65.9% of DT participants report practicing for more than 30 h per week. However, a significant majority of UG participants (95.5%) report that studying causes stress, compared to only 73.1% of DT participants reporting that work caused stress.

**Table 1:** General characteristics and work-related factors classified by dental education level (*N* = 842)

Variables	Total *n* (%)	Dental education level			*P*-value
		UG *n* (%)	PG *n* (%)	DT *n* (%)	
**Gender**					0.026*
Male	234 (27.8)	87 (33.0)	55 (22.3)	92 (27.8)	
Female	608 (72.2)	177 (67.1)	192 (77.7)	239 (72.2)	
**Age (year)**					< 0.001*
≤30	671 (79.7)	259 (98.1)	216 (87.5)	196 (59.2)	
>30	171 (20.3)	5 (1.9)	31 (12.6)	135 (40.8)	
**Medical disease**					0.058
No	656 (77.9)	219 (83.0)	186 (75.3)	251 (75.8)	
Yes	186 (22.1)	45 (17.6)	61 (24.8)	80 (24.2)	
Gastrointestinal disease	101 (12.0)	23 (8.7)	39 (15.8)	39 (11.8)	
Other disease	85 (10.1)	22 (8.3)	22 (8.9)	41 (12.4)	
**Nutritional status**					0.736
Under weight	157 (18.7)	56 (21.2)	43 (17.4)	58 (17.5)	
Normal weight	566 (67.2)	170 (64.4)	171 (69.2)	225 (68.0)	
Pre-obesity and obesity	119 (14.1)	38 (14.4)	33 (13.4)	48 (14.5)	
**Alcohol consumption (time)**					0.071
Never	213 (25.3)	59 (22.4)	62 (25.1)	92 (27.8)	
≤Once per month	489 (58.1)	171 (64.8)	142 (57.5)	176 (53.2)	
>2–4 per month	140 (16.6)	34 (12.9)	43 (17.4)	63 (19.0)	
**Hours to sleep at night**					< 0.001*
≤7	474 (56.3)	160 (60.6)	180 (72.9)	134 (40.5)	
>7	368 (43.7)	104 (39.4)	67 (27.1)	197 (59.5)	
**Hours to exercise per week**					0.102
No exercise	308 (36.6)	104 (39.4)	96 (38.9)	108 (32.6)	
≤150 min	440 (52.3)	131 (49.6)	132 (53.4)	177 (53.5)	
>150 min	94 (11.2)	29 (11.0)	19 (7.7)	46 (13.9)	
**Hours for dental practice per week**					< 0.001*
<11 h	140 (16.6)	81 (30.7)	39 (15.8)	20 (6.0)	
11–20 h	169 (20.1)	65 (24.6)	72 (29.2)	32 (9.7)	
21–30 h	270 (32.1)	117 (44.3)	92 (37.3)	61 (18.4)	
>30 h	263 (31.2)	1 (0.4)	44 (17.8)	218 (65.9)	
**Studying/Working cause stress**					< 0.001*
No	166 (19.7)	12 (4.6)	65 (26.3)	89 (26.9)	
Yes	676 (80.3)	252 (95.5)	182 (73.7)	242 (73.1)	

* The significance level was taken at *P* < 0.05

Regarding the environmental-related factors (Table 2), approximately one-third of participants across all groups resided in their own house or condominium (UG: 27.3%, PG: 29.2%, DT: 35.1%), while a similar proportion lived in a family house (UG: 27.3%, PG: 28.3%, DT: 34.1%). Notably, the majority in the UG (55.7%) and DT (68.6%) groups reported living with others, except for the PG group, where 54.7% reported living alone. Similarly, nearly equal proportions across all groups reported their families regularly consumed nutritious meals (UG: 62.1%, PG: 62.8%, DT: 69.8%). However, fewer respondents in the UG (39.8%) and PG (42.1%) groups reported consistent consumption of nutritious meals by their partners or loved ones, contrasting with the majority in the DT group (52.3%). Most participants across all groups reported regular exposure to media or resources about healthy eating (UG: 70.5%, PG: 74.9%, DT: 80.1%). The presence of stores selling healthy food in nearby residential areas was affirmed by the majority of respondents across all groups (UG: 62.9%, PG: 65.6%, DT: 64.1%), as well as at their study or work locations (UG: 66.7%, PG: 72.5%, DT: 52.9%). However, only about half of the respondents perceived food prices at nearby health food stores as affordable (UG: 48.9%, PG: 56.3%, DT: 58.9%).

**Table 2:** Environmental-related information of participants classified by Dental education level

Environmental-related information	Total *n* (%)	Dental education level			*P*-value
		UG *n* (%)	PG *n* (%)	DT *n* (%)	
**Current living place**					0.004*
Own house or condominium	260 (30.9)	72 (27.3)	72 (29.2)	116 (35.1)	
Family house	255 (30.3)	72 (27.3)	70 (28.3)	113 (34.1)	
Rental property	327 (38.8)	120 (45.5)	105 (42.5)	102 (30.8)	
**With whom are you currently residing?**					< 0.001*
Living alone	356 (42.3)	117 (44.3)	135 (54.7)	104 (31.4)	
Living with other people	486 (57.7)	147 (55.7)	112 (45.3)	227 (68.6)	
**The family regularly consumes nutritious meals**					0.089
No	292 (34.7)	100 (37.9)	92 (37.3)	100 (30.2)	
Yes	550 (65.3)	164 (62.1)	155 (62.8)	231 (69.8)	
**Your partner or beloved consistently consumes nutritious meals**					0.005*
No	460 (54.6)	159 (60.2)	143 (57.9)	158 (47.7)	
Yes	382 (45.4)	105 (39.8)	104 (42.1)	173 (52.3)	
**The adjacent residence provides access to healthful food options for purchase**					0.815
No	302 (35.9)	98 (37.1)	85 (34.4)	119 (36.0)	
Yes	540 (64.1)	166 (62.9)	162 (65.6)	212 (64.1)	
**The adjacent studying/working place provides access to healthful food options for purchase**					< 0.001*
No	312 (37.1)	88 (33.3)	68 (27.5)	156 (47.1)	
Yes	530 (63.0)	176 (66.7)	179 (72.5)	175 (52.9)	
**Do you regularly encounter media or resources about healthy eating?**					0.025*
No	206 (24.5)	78 (29.6)	62 (25.1)	66 (19.9)	
Yes	636 (75.5)	186 (70.5)	185 (74.9)	265 (80.1)	
**The health food stores you encounter offer affordable food prices.**					0.044*
No	379 (45.0)	135 (51.1)	108 (43.7)	136 (41.1)	
Yes	463 (55.0)	129 (48.9)	139 (56.3)	195 (58.9)	

* The significance level was taken at *P* < 0.05

### Dietary behaviors (practice)

The dietary behaviors of dental professionals are summarized in Table 3. Most participants reported occasional consumption of all five main food groups (UG: 53.4%, PG: 43.7%, DT: 40.2%) and frequent ordering of vegetable-based dishes (UG: 56.1%, PG: 65.2%, DT: 57.7%). A significant majority also indicated they often avoided high-fat, sweetened, or salty foods (UG: 51.5%, PG: 62.8%, DT: 58.6%), as well as partially cooked or raw foods (UG: 53.0%, PG: 67.2%, DT: 59.8%). Across all groups, more than two-thirds often refrained from consuming fermented foods and alcoholic beverages (UG: 67.4%, PG: 68.8%, DT: 68.9%). About half reported avoiding food less than 3 h before bedtime (UG: 47.0%, PG: 54.3%, DT: 50.2%). Interestingly, nearly 5% of UG reported never eating on time, while about one-third reported eating on time sometimes (33.3%), whereas over half of PG (56.7%) and DT (52.6%) often do. Less than half of UG respondents (47%) reported sometimes avoiding snacks or sweet desserts between meals, while more than half of PG (50.6%) and DT (56.8%) participants often did. Lastly, approximately half of UG and PG participants often avoided fast food items like hamburgers, French fries, pizza, and fried chicken, while a majority of DT participants reported the same behavior (UG: 52.3%, PG: 57.1%, DT: 64.7%).

**Table 3:** Dietary behaviors classified by dental education level

Items	Never *n* (%)	Sometime *n* (%)	Often *n* (%)	Regularly *n* (%)	Mean (SD)
**Practice question 1: You regularly consume all five main food groups**					
UG	11 (4.2)	141 (53.4)	81 (30.7)	31 (11.7)	1.5 (0.76)
PG	9 (3.6)	108 (43.7)	103 (41.7)	27 (10.9)	1.6 (0.73)
DT	6 (1.8)	133 (40.2)	128 (38.7)	64 (19.3)	1.8 (0.78)
**Practice question 2: You order food with vegetables.**					
UG	21 (8.0)	27 (10.2)	68 (25.8)	148 (56.1)	2.3 (0.95)
PG	5 (2.0)	25 (10.1)	56 (22.7)	161 (65.2)	2.5 (0.76)
DT	15 (4.5)	35 (10.6)	90 (27.2)	191 (57.7)	2.4 (0.85)
**Practice question 3: You do not consume high fat, sweetened, or salty foods.**					
UG	12 (4.6)	97 (36.7)	136 (51.5)	19 (7.2)	1.6 (0.69)
PG	4 (1.6)	72 (29.2)	155 (62.8)	16 (6.5)	1.7 (0.60)
DT	13 (3.9)	93 (28.1)	194 (58.6)	31 (9.4)	1.7 (0.68)
**Practice question 4: You do not consume partially cook or raw food.**					
UG	6 (2.3)	59 (22.4)	140 (53.0)	59 (22.4)	2.0 (0.73)
PG	4 (1.6)	33 (13.4)	166 (67.2)	44 (17.8)	2.0 (0.61)
DT	7 (2.1)	49 (14.8)	198 (59.8)	77 (23.3)	2.0 (0.68)
**Practice question 5: You do not consume fermented food and drinking alcoholic beverages.**					
UG	4 (1.5)	34 (12.9)	178 (67.4)	48 (18.2)	2.0 (0.61)
PG	4 (1.6)	34 (13.8)	170 (68.8)	39 (15.8)	2.0 (0.60)
DT	10 (3.0)	35 (10.6)	228 (68.9)	58 (17.5)	2.0 (0.63)
**Practice question 6: You avoid consuming meals less than 3 h before going to bed.**					
UG	27 (10.2)	78 (29.6)	124 (47.0)	35 (13.3)	1.6 (0.84)
PG	20 (8.1)	61 (24.7)	134 (54.3)	32 (13.0)	1.7 (0.79)
DT	31 (9.4)	90 (27.2)	166 (50.2)	44 (13.3)	1.7 (0.82)
**Practice question 7: You consume food on time.**					
UG	13 (4.9)	88 (33.3)	125 (47.4)	38 (14.4)	1.7 (0.77)
PG	5 (2.0)	76 (30.8)	140 (56.7)	26 (10.5)	1.8 (0.66)
DT	10 (3.0)	76 (23.0)	174 (52.6)	71 (21.5)	1.9 (0.75)
**Practice question 8: You avoid consuming snacks or sweet desserts between meals.**					
UG	36 (13.6)	124 (47.0)	97 (36.7)	7 (2.7)	1.3 (0.73)
PG	16 (6.5)	102 (41.3)	125 (50.6)	4 (1.6)	1.5 (0.64)
DT	15 (4.5)	114 (34.4)	188 (56.8)	14 (4.2)	1.6 (0.64)
**Practice question 9: You avoid consuming fast food such as hamburgers, French fries, pizza, and fried chicken.**					
UG	15 (5.7)	109 (41.3)	138 (52.3)	2 (0.8)	1.5 (0.62)
PG	9 (3.6)	97 (39.3)	141 (57.1)	0 (0.0)	1.5 (0.57)
DT	22 (6.7)	90 (27.2)	214 (64.7)	5 (1.5)	1.6 (0.63)

#### Knowledge, attitude, and practice scores and their correlations

**Fig. 1:**
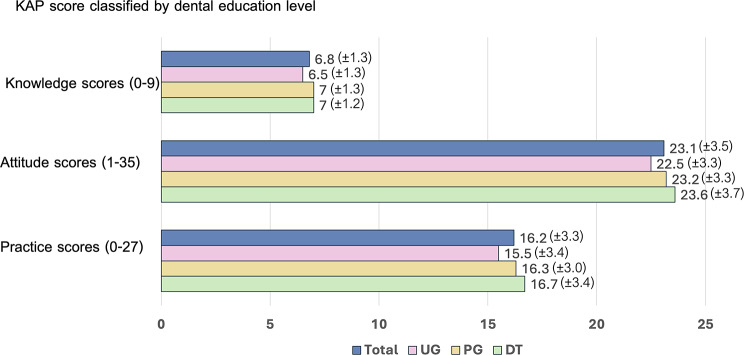
KAP score classified by dental education level

The average knowledge score for all participants was approximately 6.8 out of 9 (SD = 1.3). In terms of attitude and practice related to healthy dietary behaviors, the mean scores are 23.1 out of 35 (SD = 3.5) and 16.2 out of 27 (SD = 3.3), respectively. Specifically, dental undergraduates (UG) had slightly lower knowledge scores (Mean = 6.5, SD = 1.3), compared to postgraduates (Mean = 6.5, SD = 1.3) and dentists (Mean = 7.0, SD = 1.2). This trend remained consistent across attitude and practice scores, as demonstrated in Fig. 1.

Considering the correlations among knowledge, attitude and practice among each group of dental professionals, knowledge demonstrated a positive weak correlation solely with practices in the PG group (*r* = 0.213, *P*-value < 0.001). However, attitude correlated with practice in all groups (UG, PG, and DT) with respective values of *r* = 0.485, 0.549, and 0.672, with all *P*-values < 0.001 (Table 4).

**Table 4:** Correlations of knowledge, attitude, and practice classified by dental education level

Variable	Attitude		Practice	
	*r*	*P*-value	*r*	*P*-value
UG group				
Knowledge	0.189	0.002*	0.055	0.371
Attitude	-	-	0.485	< 0.001*
***PG group***				
Knowledge	0.121	0.057	0.213	< 0.001*
Attitude	-	-	0.549	< 0.001*
***DT group***				
Knowledge	0.048	0.387	0.031	0.572
Attitude	-	-	0.672	< 0.001*

* The significance level was taken at *P* < 0.05

### Associations of determinants and healthy dietary behaviors in dental professionals

Multivariate linear regression revealed significant associations between healthy dietary behaviors and their determinants. Notably, attitude emerged as a predominant factor, exhibiting the strongest positive association with healthy dietary behaviors across all groups (UG: ß = 0.370, PG: ß = 0.512, DT: ß = 0.642;

*P*-value < 0.001). Conversely, alcohol consumption consistently showed a negative association with healthy dietary behaviors among participants (UG: ß = -0.135, PG: ß = -0.220, DT: ß = -0.216; *P*-value < 0.001).

Upon subgroup analysis, distinct associations emerged (Tables 5, 6 and 7). In the UG group, family consumption of nutritious meals (ß = 0.114; *P*-value = 0.038) and cohabitation with someone (ß = 0.113; *P*-value = 0.032) demonstrated relevance, alongside the positive association between attitude and healthy dietary behaviors. Additionally, there was a negative association with the factor of stress while studying or working (ß = -0.123; *P*-value = 0.02). In contrast, the PG group revealed that knowledge (ß = 0.143, *P*-value = 0.006) was a significant determinant associated with healthy dietary behaviors.

**Table 5:** Multivariate regression result for factors influencing dietary behaviors among undergraduate (UG) group (*n* = 264)

Variables	ß	95% CI	*P*-value
Attitude scores	0.370	(0.26, 0.49)	< 0.001
Hours to exercise per week	0.184	(0.40, 1.53)	< 0.001
Alcohol consumption	-0.135	(-1.39, -0.18)	0.011
Studying/Working cause stress	-0.123	(-3.70, -0.33)	0.020
With whom are you currently residing?	0.113	(0.07, 1.48)	0.032
The family regularly consumes nutritious meals	0.114	(0.05, 1.55)	0.038

* The significance level was taken at *P* < 0.05. Adjusted R-squared = 0.301

**Table 6:** Multivariate regression result for factors influencing dietary behaviors among postgraduate (PG) group (*n* = 247)

Variables	ß	95% CI	*P*-value
Attitude scores	0.512	(0.37, 0.55)	< 0.001
Alcohol consumption	-0.220	(-1.48, -0.56)	< 0.001
Knowledge scores	0.143	(0.10, 0.58)	0.006

* The significance level was taken at *P* < 0.05. Adjusted R-squared = 0.375

**Table 7:** Multivariate regression result for factors influencing dietary behaviors among Dentist (DT) group (*n* = 331)

Variables	ß	95% CI	*P*-value
Attitude scores	0.642	(0.52, 0.66)	< 0.001
Alcohol consumption	-0.216	(-1.47, -0.69)	< 0.001

* The significance level was taken at *P* < 0.05. Adjusted R-squared = 0.484

## Discussion

Research into dentists’ dietary behaviors often emphasizes their pivotal role in promoting healthy dietary habits among patients. However, most studies primarily assess dentists’ nutrition knowledge, overlooking other influential factors that shape eating behaviors [28, 33], thereby oversimplifying the complex determinants of dietary habits. In contrast, our study is distinctive in its inclusion of three groups of dentists with varying levels of education and experience. This approach provides a comprehensive perspective on how eating habits evolve over time. The Knowledge, Attitude, and Practice (KAP) model was employed to systematically analyzed the factors influencing dentists’ eating habits across different educational and professional stages.

A majority of participants reported good health and low alcohol consumption. However, a significant proportion faced challenges related to insufficient sleep and inadequate physical activity levels. Although dental professionals generally demonstrated healthy dietary practices, disparities were evident in meal timing habits, particularly among undergraduate students. This disparity may arise from the demanding nature of academic workload, as evidenced by previous studies investigating factors influencing eating habits among university students [34]. Moreover, UG participants expressed significantly higher levels of stress attributed to studying/work compared to PG and DT groups. This observation aligns with previous research indicating that academic demands induce stress and anxiety [35, 36]. Such findings may suggest a perceived lack of control, where dental students experience uncertainty regarding their academic and professional lives, particularly during intense periods of study and clinical training, thereby elevating stress levels [37]. Understanding these variations is vital for developing targeted interventions aimed at fostering healthier eating habits and overall well-being among dental professionals.

The KAP model has frequently served as a theoretical framework for elucidating human behavior, proposing that the acquisition of knowledge leads to a favorable attitude conducive to the adoption of practice [38]. While previous studies have shown significant correlations between knowledge and healthier dietary choices [39], the findings in this research indicated a weak association between knowledge and healthy dietary behaviors, particularly in the PG group. This could be a result of other internal and external factors, including individual motivations, environmental factors, and situational constraints, which could significantly impact the behaviors [40]. Consequently, it is imperative to engage in a comprehensive examination of these factors to discern their implications in shaping dietary behaviors.

The dietary behaviors of dentists were significantly influenced by their attitude and alcohol consumption. Through multiple linear regression analysis, attitude emerged as the predominant factor influencing dietary behaviors across various groups of participants. Consistent with prior research [41], it is evident that attitudes and motivations regarding the consumption of healthy foods directly correlate with lifestyle choices and dietary patterns. The subsequent significant factor influencing dietary behaviors is alcohol consumption, which can exert contrasting effects on dietary behavior patterns. Regular alcohol intake has been associated with unhealthy practices such as smoking, sedentary behaviors, and erratic eating routines, all of which contribute to poor dietary behaviors. Current evidence indicates that alcohol consumption, particularly of beer and spirits, is correlated with diminished intake of nutritious foods, lower dietary quality, and the adoption of unhealthy lifestyle behaviors such as smoking and physical inactivity [42].

When examining the determinants associated with healthy eating behaviors among dental professionals across diverse educational backgrounds, there were discrepancies in the factors influencing dietary habits among these cohorts. Firstly, the UG group may encounter heightened levels of stress attributable to academic demands [43], thereby enhancing the influence of stress-related factors on their dietary behaviors in comparison to the PG and DT groups. Additionally, the impact of knowledge on dietary behaviors may exhibit variability across these groups owing to differences in their exposure to nutrition education and awareness. The PG group may possess higher level of nutrition knowledge, leading to a stronger association between knowledge acquisition and dietary behaviors. Available evidence has revealed the correlation between education levels and dietary behaviors, highlighting that individuals with higher educational levels tend to exhibit a propensity towards consuming more nutritious foods [40]. Although additional factors may contribute to these differences, they are not exclusive determinants therein in this research.

Despite the rigorous design aimed at ensuring the validity and reliability of this research, it is necessary to discuss a couple potential limitations. As the data collection was based on an online survey, there could be response biases associated with self-reporting. The accuracy of the data relied upon respondents’ honesty and comprehension, thereby raising concerns of both overestimation and underestimation in the findings [44, 45]. Additionally, due to the diverse formats of our items in each construct, which encompassed both checklist and scale questions, Confirmatory Factor Analysis (CFA) cannot be performed to assess item construct validity [46]. Another concern was the age distribution of participants, with a majority falling under 30 years old. This demographic data may not accurately reflect the older segment of dental professionals, potentially impacting the generalizability of findings. However, it is essential to note that age did not significantly influence the dietary behaviors of DT respondents.

As this study highlights the significance of both attitude and knowledge in promoting healthy dietary behaviors among dental professionals, future research should investigate interventions addressing the underlying attitudes and motivations driving dietary decisions. Embracing an approach that combines attitude development alongside knowledge dissemination may hold promise in fostering sustainable behavior change and improving the overall health and wellness of dental professionals in the long term.

## Conclusion

The findings retrieved from this research revealed significant variations in dietary behaviors across diverse educational levels of dental professionals. Attitude emerged as the predominant factor influencing dietary behaviors, while a weak association between knowledge and healthy dietary behaviors was observed, emphasizing the importance of targeting attitudes toward diet in intervention efforts. Tailored strategies should be considered to address the distinct challenges encountered by dental professionals at various career stages, contributing to the development of interventions to enhance dietary practices and overall well-being.

### Electronic supplementary material

Below is the link to the electronic supplementary material.

Supplementary Material 1

## Data Availability

The data that support the findings of this study are available from the corresponding author, up-on reasonable request. The data are not publicly available due to information that could compromise the privacy of research participants.
