# Supplementary material for: Determinants of dietary behaviors among dental professionals: insights across educational levels
Source: BMC Oral Health. 2024 Jun 24;24:724. doi: 10.1186/s12903-024-04502-4 (PMC11197208; doi:10.1186/s12903-024-04502-4)
Supplement: Supplementary file 1 — Supplementary Material 1 [file 12903_2024_4502_MOESM1_ESM.docx]

**Supplement table 1** shows knowledge of all participants classified by participant education level
(n: UG=264, PG=247 and DT=331)

| **Items** | **Right answer  n (%)** | **I don’t know  n (%)** | **Wrong answer n (%)** | **Mean (SD)** |
| --- | --- | --- | --- | --- |
| **Knowledge question 1: Individuals should aim to consume all six food groups (including the five main food groups) while also paying attention to their body weight and waist circumference** | | | | |
| UG | 246 (93.2) | 7 (2.6) | 11 (4.2) | 0.9 (0.25) |
| PG | 241 (97.6) | 5 (2) | 1 (0.4) | 1.0 (0.15) |
| DT | 321 (97) | 4 (1.2) | 6 (1.8) | 1.0 (0.17) |
| **Knowledge question 2: Individuals should consume polished rice and focus on consuming mainly starchy-rich foods.** | | | | |
| UG | 162 (61.4) | 15 (5.7) | 87 (32.9) | 0.6 (0.49) |
| PG | 177 (71.6) | 14 (5.7) | 56 (22.7) | 0.7 (0.45) |
| DT | 249 (75.2) | 17 (5.2) | 65 (19.6) | 0.8 (0.43) |
| **Knowledge question 3: People of working age should aim to have 6 servings of meat per meal.** | | | | |
| UG | 20 (7.6) | 134 (50.7) | 110 (41.7) | 0.1 (0.27) |
| PG | 29 (11.7) | 125 (50.6) | 93 (37.7) | 0.1 (0.32) |
| DT | 32 (9.7) | 169 (51) | 130 (39.3) | 0.1 (0.30) |
| **Knowledge question 4: Individuals should prioritize consuming ample vegetables, regularly incorporate fruits of different colors into their diet, and be mindful to avoid excessively sweet fruits.** | | | | |
| UG | 256 (96.6) | 4 (1.7) | 4 (1.7) | 1.0 (0.17) |
| PG | 238 (96.4) | 6 (2.4) | 3 (1.2) | 1.0 (0.19) |
| DT | 326 (98.5) | 1 (0.3) | 4 (1.2) | 1.0 (0.12) |
| **Knowledge question 5: Individuals should consume unsweetened milk and also obtain calcium from other types of foods.** | | | | |
| UG | 253 (95.8) | 7 (2.7) | 4 (1.5) | 1.0 (0.20) |
| PG | 225 (91.1) | 12 (4.9) | 10 (4) | 0.9 (0.29) |
| DT | 312 (94.3) | 8 (2.4) | 11 (3.3) | 0.9 (0.23) |
| **Knowledge question 6: Individuals can prepare their meals by adding 3 teaspoons of sugar and 2 teaspoons of fish sauce to each serving.** | | | | |
| UG | 119 (45.1) | 113 (42.8) | 32 (12.1) | 0.5 (0.50) |
| PG | 160 (64.8) | 72 (29.1) | 15 (6.1) | 0.6 (0.48) |
| DT | 203 (61.3) | 105 (31.7) | 23 (7) | 0.6 (0.49) |
| **Knowledge question 7: Individuals should consume clean, safe, and freshly cooked food. They should avoid eating partially cooked or raw food.** | | | | |
| UG | 257 (97.4) | 3 (1.1) | 4 (1.5) | 1.0 (0.16) |
| PG | 245 (99.2) | 0 (0.0) | 2 (0.8) | 1.0 (0.09) |
| DT | 329 (99.4) | 1 (0.3) | 1 (0.3) | 1.0 (0.08) |
| **Knowledge question 8: When individuals drink a sufficient amount of clean water, they may also consume sweetened drinks, tea, coffee, or soft drinks, with a limit of 2 servings per day.** | | | | |
| UG | 149 (56.5) | 50 (18.9) | 65 (24.6) | 0.6 (0.50) |
| PG | 173 (70) | 33 (13.4) | 41 (16.6) | 0.7 (0.46) |
| DT | 226 (68.3) | 67 (20.2) | 38 (11.5) | 0.7 (0.47) |
| **Knowledge question 9: Individuals should avoid consuming fermented foods and drinking alcohol** | | | | |
| UG | 250 (94.7) | 7 (2.7) | 7 (2.7) | 0.9 (0.22) |
| PG | 237 (96) | 2 (0.8) | 8 (3.2) | 1.0 (0.20) |
| DT | 317 (95.8) | 4 (1.2) | 10 (3) | 1.0 (0.20) |

**Supplement table 2** shows attitude of all participants classified by participant education level (n: UG=264, PG=247 and DT=331)

| **Items** | **Strongly disagree  n (%)** | **Disagree n (%)** | **Unsure  n (%)** | **Agree  n (%)** | **Strongly agree  n (%)** | **Mean (SD)** |
| --- | --- | --- | --- | --- | --- | --- |
| **Attitude question 1: Do you agree that the type of food consumed affects health, body shape, weight, or daily activities?** | | | | | | |
| UG | 0 (0.0) | 2 (0.8) | 4 (1.5) | 125 (47.4) | 133 (50.4) | 4.5 (0.57) |
| PG | 2 (0.8) | 1 (0.4) | 9 (3.6) | 84 (34.0) | 151 (61.1) | 4.5 (0.67) |
| DT | 5 (1.5) | 0 (0.0) | 2 (0.6) | 99 (29.9) | 225 (68.0) | 4.6 (0.65) |
| **Attitude question 2: You regularly pay attention to your own food consumption habits.** | | | | | | |
| UG | 5 (1.9) | 23 (8.7) | 113 (42.8) | 102 (38.6) | 21 (8.0) | 3.7 (0.79) |
| PG | 3 (1.2) | 16 (6.5) | 91 (36.8) | 122 (49.4) | 15 (6.1) | 3.5 (0.76) |
| DT | 1 (0.3) | 21 (6.3) | 95 (28.7) | 169 (51.1) | 45 (13.6) | 3.7 (0.79) |
| **Attitude question 3: Do you believe that the steps involved in preparing food or finding healthy food to consume are not difficult and do not require a lot of time?** | | | | | | |
| UG | 36 (13.6) | 131 (49.6) | 53 (20.1) | 41 (15.5) | 3 (1.1) | 2.4 (0.95) |
| PG | 43 (17.4) | 123 (49.8) | 58 (23.5) | 18 (7.3) | 5 (2.0) | 2.3 (0.90) |
| DT | 51 (15.4) | 161 (48.6) | 77 (23.3) | 32 (9.7) | 10 (3.0) | 2.4 (0.96) |
| **Attitude question 4: You dislike consuming food solely based on its taste.** | | | | | | |
| UG | 23 (8.7) | 76 (28.8) | 70 (26.5) | 81 (30.7) | 14 (5.3) | 3.0 (1.08) |
| PG | 8 (3.2) | 69 (27.9) | 56 (22.7) | 97 (39.3) | 17 (6.9) | 3.2 (1.02) |
| DT | 14 (4.2) | 95 (28.7) | 73 (22.1) | 122 (36.9) | 27 (8.2) | 3.2 (1.06) |
| **Attitude question 5: You always think about eating healthy food when you feel hungry.** | | | | | | |
| UG | 41 (15.5) | 112 (42.4) | 86 (32.6) | 24 (9.1) | 1 (0.4) | 2.4 (0.87) |
| PG | 20 (8.1) | 90 (36.4) | 96 (38.9) | 35 (14.2) | 6 (2.4) | 2.7 (0.90) |
| DT | 34 (10.3) | 112 (33.8) | 116 (35.1) | 60 (18.1) | 9 (2.7) | 2.7 (0.97) |
| **Attitude question 6: You dislike snack foods like potato chips or foods with a salty taste.** | | | | | | |
| UG | 20 (7.6) | 97 (36.7) | 59 (22.4) | 57 (21.6) | 31 (11.7) | 2.9 (1.16) |
| PG | 18 (7.3) | 83 (33.6) | 58 (23.5) | 52 (21.1) | 36 (14.6) | 3.0 (1.19) |
| DT | 27 (8.2) | 102 (30.8) | 81 (24.5) | 76 (23.0) | 45 (13.6) | 3.0 (1.19) |
| **Attitude question 7: You believe that eating a healthy breakfast is a good and necessary habit.** | | | | | | |
| UG | 11 (4.2) | 14 (5.3) | 45 (17.1) | 111 (42.1) | 83 (31.4) | 3.9 (1.03) |
| PG | 6 (2.4) | 15 (6.1) | 48 (19.4) | 85 (34.4) | 93 (37.7) | 4.0 (1.02) |
| DT | 10 (3.0) | 12 (3.6) | 55 (16.6) | 131 (39.6) | 123 (37.2) | 4.0 (0.98) |

**Supplement table 3** shows practice of all participants classified by participant education level (n: UG=264, PG=247 and DT=331)

| **Items** | **Never  n (%)** | **Sometime n (%)** | **Often  n (%)** | **Regularly n (%)** | **Mean (SD)** |
| --- | --- | --- | --- | --- | --- |
| **Practice question 1: You regularly consume all five main food groups** | | | | | |
| UG | 11 (4.2) | 141 (53.4) | 81 (30.7) | 31 (11.7) | 1.5 (0.76) |
| PG | 9 (3.6) | 108 (43.7) | 103 (41.7) | 27 (10.9) | 1.6 (0.73) |
| DT | 6 (1.8) | 133 (40.2) | 128 (38.7) | 64 (19.3) | 1.8 (0.78) |
| **Practice question 2: You order food with vegetables.** | | | | | |
| UG | 21 (8.0) | 27 (10.2) | 68 (25.8) | 148 (56.1) | 2.3 (0.95) |
| PG | 5 (2.0) | 25 (10.1) | 56 (22.7) | 161 (65.2) | 2.5 (0.76) |
| DT | 15 (4.5) | 35 (10.6) | 90 (27.2) | 191 (57.7) | 2.4 (0.85) |
| **Practice question 3: You do not consume high fat, sweetened, or salty foods.** | | | | | |
| UG | 12 (4.6) | 97 (36.7) | 136 (51.5) | 19 (7.2) | 1.6 (0.69) |
| PG | 4 (1.6) | 72 (29.2) | 155 (62.8) | 16 (6.5) | 1.7 (0.60) |
| DT | 13 (3.9) | 93 (28.1) | 194 (58.6) | 31 (9.4) | 1.7 (0.68) |
| **Practice question 4: You do not consume partially cook or raw food.** | | | | | |
| UG | 6 (2.3) | 59 (22.4) | 140 (53.0) | 59 (22.4) | 2.0 (0.73) |
| PG | 4 (1.6) | 33 (13.4) | 166 (67.2) | 44 (17.8) | 2.0 (0.61) |
| DT | 7 (2.1) | 49 (14.8) | 198 (59.8) | 77 (23.3) | 2.0 (0.68) |
| **Practice question 5: You do not consume fermented food and drinking alcoholic beverages.** | | | | | |
| UG | 4 (1.5) | 34 (12.9) | 178 (67.4) | 48 (18.2) | 2.0 (0.61) |
| PG | 4 (1.6) | 34 (13.8) | 170 (68.8) | 39 (15.8) | 2.0 (0.60) |
| DT | 10 (3.0) | 35 (10.6) | 228 (68.9) | 58 (17.5) | 2.0 (0.63) |
| **Practice question 6: You avoid consuming meals less than 3 hours before going to bed.** | | | | | |
| UG | 27 (10.2) | 78 (29.6) | 124 (47.0) | 35 (13.3) | 1.6 (0.84) |
| PG | 20 (8.1) | 61 (24.7) | 134 (54.3) | 32 (13.0) | 1.7 (0.79) |
| DT | 31 (9.4) | 90 (27.2) | 166 (50.2) | 44 (13.3) | 1.7 (0.82) |
| **Practice question 7: You consume food on time.** | | | | | |
| UG | 13 (4.9) | 88 (33.3) | 125 (47.4) | 38 (14.4) | 1.7 (0.77) |
| PG | 5 (2.0) | 76 (30.8) | 140 (56.7) | 26 (10.5) | 1.8 (0.66) |
| DT | 10 (3.0) | 76 (23.0) | 174 (52.6) | 71 (21.5) | 1.9 (0.75) |
| **Practice question 8: You avoid consuming snacks or sweet desserts between meals.** | | | | | |
| UG | 36 (13.6) | 124 (47.0) | 97 (36.7) | 7 (2.7) | 1.3 (0.73) |
| PG | 16 (6.5) | 102 (41.3) | 125 (50.6) | 4 (1.6) | 1.5 (0.64) |
| DT | 15 (4.5) | 114 (34.4) | 188 (56.8) | 14 (4.2) | 1.6 (0.64) |
| **Practice question 9: You avoid consuming fast food such as hamburgers, French fries, pizza, and fried chicken.** | | | | | |
| UG | 15 (5.7) | 109 (41.3) | 138 (52.3) | 2 (0.8) | 1.5 (0.62) |
| PG | 9 (3.6) | 97 (39.3) | 141 (57.1) | 0 (0.0) | 1.5 (0.57) |
| DT | 22 (6.7) | 90 (27.2) | 214 (64.7) | 5 (1.5) | 1.6 (0.63) |
